# Investigating genetic overlap between antidepressant and lithium response and treatment resistance in major depressive disorder

**DOI:** 10.21203/rs.3.rs-2556941/v1

**Published:** 2023-02-20

**Authors:** Yi Lu, Ying Xiong, Robert Karlsson, Jie Song, Kaarina Kowalec, Christian Rück, Robert Sigstrom, Lina Jonsson, Caitlin Clements, Evelyn Andersson, Julia Boberg, Cathryn Lewis, Patrick Sullivan, Mikael Landén

**Affiliations:** Karolinska Institutet; Karolinska Institutet; Karolinska Institutet; Karolinska Institutet; Karolinska Institutet; Karolinska Institutet; University of Pennsylvania; University of Pennsylvania; University of Pennsylvania; King’s College London; King’s College London; King’s College London; University of North Carolina; Gothenburg University

## Abstract

Treatment response and resistance in major depressive disorder (MDD) are suggested to be heritable. Due to significant challenges in defining treatment-related phenotypes, our understanding of their genetic bases is limited. This study aimed to derive a stringent definition of treatment resistance and to investigate genetic overlap between treatment response and resistance in MDD. Using electronic medical records on the use of antidepressants and electroconvulsive therapy (ECT) from Swedish registers, we derived the phenotype of treatment-resistant depression (TRD) within ~ 4 500 individuals with MDD in three Swedish cohorts. Considering antidepressants and lithium are first-line treatment and augmentation used for MDD, respectively, we generated polygenic risk scores of antidepressant and lithium response for individuals with MDD, and evaluated their associations with treatment resistance by comparing TRD with non-TRD. Among 1 778 ECT-treated MDD cases, nearly all (94%) used antidepressants before first ECT, and the vast majority had at least one (84%) or two (61%) antidepressants of adequate duration, suggesting these MDD cases receiving ECT were resistant to antidepressants. We found that TRD cases tend to have lower genetic load of antidepressant response than non-TRD, although the difference was not significant; furthermore, TRD cases had significantly higher genetic load of lithium response (OR = 1.10–1.12 under different definitions). The results support evidence of heritable components in treatment-related phenotypes and highlight the overall genetic pro le of lithium-sensitivity in TRD. This finding further provides a genetic explanation for lithium efficacy in treating TRD.

## Introduction

Major depressive disorder (MDD) is a leading cause of disability worldwide, and associated with a staggering burden for affected individuals and the society^1, 2^. Antidepressants are a first-line treatment for MDD, and generally effective in reducing symptoms and preventing relapse^3^. However, only one-third of individuals with MDD reach complete symptom remission, while another one-third or more fail to respond to antidepressant medications^4^. When individuals with MDD experience insufficient response to first-line antidepressants, other treatment strategies might be used to enhance treatment effect, including antidepressant combinations or switches, or augmentation therapies with lithium or atypical antipsychotics^5^. Lithium, which is primarily used to prevent mood episodes in bipolar disorder but also used to augment antidepressants, has been shown to prevent relapse and hospital re-admission in MDD^6,7^. In addition, electroconvulsive therapy (ECT) is typically recommended as second- or third-line treatment for individuals with severe MDD who are not responsive to pharmacotherapy, psychotherapy, or have an urgent need for rapid clinical improvement in mood due to psychotic symptoms or suicidality^8, 9^.

It has been hypothesized that individual variation in response to medications used to treat MDD may have a genetic component. For treatment response, the largest genome-wide association study (GWAS) to-date on antidepressant response (N = 5 218) led by the MDD working group of the Psychiatric Genomics Consortium estimated that 13.2% (95% CI = 2.2–24.2%) of variance in antidepressant response, measured by symptom remission, was explained by common genetic variants (*i.e*., SNP-based heritability)^10^. This study further demonstrated genetic overlap of antidepressant response with risk for schizophrenia^10^. The International Consortium on Lithium Genetics (ConLi^+^Gen) conducted a GWAS of lithium response in 2 563 individuals with bipolar disorder^11^. Although the SNP-based heritability was not reported, this study identified genetic markers associated with a region containing long non-coding RNAs^11^. Follow-up studies also suggested that higher genetic loading for certain psychiatric disorders (MDD and schizophrenia) were associated with worse lithium response in bipolar disorder, providing evidence for shared genetics between lithium response and risks for psychiatric disorders^12, 13^. The term of treatment resistance is used when an individual fails to respond to adequate treatments^14^. Comparing treatment-resistant depression (TRD) and non-TRD cases, the findings from previous studies generally supported that MDD treatment resistance has a genetic component and shares genetic risks with certain psychiatric disorders^15–18^. For example, a recent study based on UK primary care records estimated the SNP-based heritability of treatment resistance at 7.7% (95% CI = 2.4–13.0% in observed scale) and demonstrated that the polygenicity of ADHD was associated with treatment resistance among MDD^16^.

Nonetheless, significant challenges remain in defining treatment resistance in psychiatric research^14, 19,20^. For MDD, typically only the use of antidepressant medications is considered in defining TRD, and the required number of failed antidepressant trials has been debated^14–17, 19^. Unclear definition of TRD makes comparisons across studies difficult and limits our understanding of biological underpinnings in MDD treatment resistance. Here, we defined TRD based on both antidepressant and ECT use, given that ECT is indicated for individuals with severe MDD who fail to respond to first-line treatment^21, 22^. We also leveraged the latest GWAS of antidepressants and lithium response to investigate the genetic overlap between treatment response and resistance in three Swedish cohorts with over 4 500 individuals with MDD.

## Materials And Methods

### Data source

Study population. This study consisted of case-only samples with clinical diagnoses of MDD. We extracted MDD cases from three Swedish cohorts. First, we used the Predictors for ECT (PREFECT) study that recruited individuals from the Swedish National Quality Register for ECT between 2013 and 2017^23^. From the PREFECT study, we extracted the severe MDD cases, *i.e*., those receiving ECT for a major depressive episode in the context of MDD, but excluding cases receiving ECT for other mood disorders like bipolar or schizoaffective disorder (N = 1 922)^24^. Second, we used the Internet-based Cognitive Behavior Therapy (iCBT) study which recruited mild-moderate MDD cases who were treated with internet-based cognitive behavior therapy (N = 964)^25^. Third, we used the population-based cohort of the Swedish Twin Studies of adults: Genes and Environment (STAGE), from which we extracted 1 686 MDD cases who either had a clinical diagnosis of MDD from the linked patient registers or fulfilled DSM-IV diagnostic criteria, and had no diagnosis of schizophrenia and bipolar disorder^26^. All participants provided informed consent. The studies were approved by the Regional Ethics Review Board in Stockholm. Further details about these samples have been described previously^24–26^. Altogether, 4 572 MDD cases were eligible for the study.

GWAS summary statistics of antidepressant response. GWAS summary statistics of antidepressant response were obtained from the largest GWAS of antidepressant response to-date (N = 5 218 MDD cases)^10^. Two phenotypes of antidepressant response were assessed in the GWAS. The first phenotype defined remission as a binary trait, *i.e*., “whether depressive symptom score in the rating scale decreases to a pre-specified threshold after antidepressant use” (“remission”). The second phenotype was a quantitative trait, defined as “the percentage change of symptoms scale after antidepressant use” (referred as “percentage improvement”), with a higher percentage improvement indicating a better treatment response. Since the significant SNP-based heritability was reported only for the binary “remission” phenotype^10^, we used the summary statistics of this phenotype in all analyses.

GWAS summary statistics of lithium response. GWAS summary statistics of lithium response were obtained from the largest GWAS on lithium response in patients with bipolar disorder (N = 2 563) conducted by the ConLi^+^Gen Consortium^11, 27^. Similar to antidepressant response GWAS, two phenotypes of lithium response were assessed: a binary trait dichotomized by a pre-defined cutoff of the rating scale (good vs. poor response to lithium treatment) and a quantitative measure of symptom improvement; here we also used summary statistics of the binary trait and based on samples with European ancestry (N = 2 343) in all analyses.

GWAS summary statistics of psychiatric disorders. We also obtained GWAS summary statistics of corresponding psychiatric disorders (MDD and bipolar disorder) from the latest published GWAS (N = 500 199 in MDD GWAS and N = 51 710 in bipolar disorder GWAS) for sensitivity analyses^28, 29^.

### Phenotype definitions

TRD. We derived TRD definitions in the PREFECT samples who have received ECT in the context of MDD. Repeated treatment failure is one frequent reason for administering ECT in individuals with MDD^21, 22^, but ECT is also used as the first-line treatment in cases of severe psychotic symptoms or life-threatening conditions^30^. Therefore, we utilized the prescription records of antidepressants before the first ECT treatment to ensure our definitions capture actual TRD cases, *i.e*., individuals with MDD who have received ECT due to treatment resistance. The drug prescription records were obtained from the Swedish National Prescription Drug Register (PDR, available between July 2005 to May 2018)^24^, using Anatomical Therapeutic Chemical (ATC) codes under the category of “N06A” for antidepressants and N05AN01 for lithium. We defined TRD cases in the PREFECT samples as those who have used at least one (“*narrow_1*” TRD definition) or two different antidepressants (“*narrow_2*” TRD) of adequate duration before the first ECT treatment. The treatment duration for each antidepressant was calculated based on the first and last dispense date of the antidepressant in the same treatment period (*i.e*., the time interval between two consecutive prescriptions for the same antidepressant within 120 days^31^). Similar to other studies, we considered adequate treatment duration of ≥ 6 consecutive weeks in order to account for the expected length of therapeutic effect and to distinguish from drug switches due to adverse effects^16, 32^. For comparison, we also considered a “*broad definition*” without restriction for antidepressant use before the first treatment of ECT.

Non-TRD. The comparison group of non-TRD cases was derived from iCBT and STAGE samples. To match the broad definition of TRD, we considered those without ECT as non-TRD. Since the iCBT samples were mild-moderate MDD cases recruited to begin internet-based cognitive behavior therapy^25^, these samples were unlikely to be treated with ECT. For the STAGE samples, we obtained medication data from the PDR to de ne adequate antidepressant duration similarly to our definition in TRD. These STAGE samples were also linked with the patient register from which we identified 19 individuals who had received ECT. In STAGE, non-TRD cases were defined as MDD cases who have used antidepressants but with no more than two antidepressants of adequate duration (≥ 6 weeks), and have never used ECT. This definition was chosen to capture likely antidepressant responders and to correspond with the commonly used cut-off in the literature for defining TRD/non-TRD cases^19^.

Taken together, we derived three sets of comparisons, with one broad definition based on ECT use only and two narrow definitions integrating both ECT and antidepressant use:

1. *Broad*: MDD cases receiving ECT treatment (TRD), compared with those without ECT (non-TRD);
2. *Narrow_1*: MDD cases with ≥ 1 antidepressant of adequate duration before the first ECT (TRD), compared with those with ≤ 2 antidepressants of adequate duration and without ECT (non-TRD);
3. *Narrow_2*: MDD cases with ≥ 2 different antidepressants of adequate duration before the first ECT (TRD), compared with those with ≤ 2 antidepressants of adequate duration and without ECT (non-TRD).

### Genotyping, quality control and imputation

Genotyping was conducted at Life and Brain GmbH (Bonn, Germany) for both PREFECT and iCBT samples, using Illumina Infinium Global Screening Arrays (v1)^24, 25^. Samples from STAGE were genotyped with the same array by the SNP&SEQ Technology Platform (Uppsala, Sweden)^26^.

After harmonizing the markers and allele coding, we merged raw genotype data from the three studies. Before merging, we excluded SNPs from each study for the following reasons: monomorphic sites; indels; strand ambiguous; minor allele frequency (MAF) < 0.01. In the merged data, 4 187 MDD cases had both genotypes and phenotypes. We then used the PGC Ricopili pipeline for quality control (QC)^33^. After first removing SNPs with missingness > 5%, we removed 14 samples (0.33%) due to any of the following criteria: per-sample call rate < 0.98; excessive heterozygosity (FHET outside +/− 0.20); or sex mismatch. We excluded SNPs due to any of the following: per-SNP call rate < 0.98; invariant; Hardy-Weinberg disequilibrium (P < 1e^− 6^ in controls and cases separately); difference in call rate between cases and controls > 0.01; MAF < 0.01. We retained 459 906 SNPs (92.11%) after QC. By projecting the first two principal components (PCs) of the study samples to the reference panel of the 1000 Genome global population, we identified and excluded 56 (1.34%) non-European ancestral outliers whose first two PCs exceeded 6 standard deviations from the mean values of the European samples in the reference population. Relatedness was estimated from the genotype data and one in each pair of related individuals (75 pairs with $\hat{\pi }>0.2$; $\hat{\pi }$ is estimated proportion of the genome shared identical-by-descent) was excluded.

After QC, Sanger imputation service was used to impute genotype data to the reference panel of Haplotype Reference Consortium data (HRC1.1). EAGLE2 and IMPUTE2 were used for pre-phasing and imputation, respectively^34–36^.

### Statistical analyses

Polygenic risk scores (PRS). Before calculating the PRS, we further excluded the following SNPs from GWAS summary statistics: 1) SNPs with MAF < 0.1 or INFO score < 0.9; 2) duplicate SNPs; 3) strand-ambiguous SNPs; 4) SNPs in major histocompatibility complex regions (chr6:28–34 Mb). SNPs overlapping with Hapmap3 were extracted and the summary statistics were rescaled to account for linkage disequilibrium (LD) in SBayesR, which is a state-of-the-art method with high prediction accuracy in psychiatric disorders^37, 38^. Finally, PRS was calculated in imputed data for each individual as the sum of the number of risk alleles weighted by allelic effects in PLINK (version 2.0)^39^; and standardised within the whole sample. We also calculated the PRS of MDD and bipolar disorder in the same way for sensitivity analyses.

Association analysis. To examine the genetic association of treatment response with TRD status, we first tested the mean differences in the PRS of antidepressant or lithium response among TRD cases compared to non-TRD cases (t-test). Logistic regression was used to estimate the odds ratios (OR) corresponding to per standard deviation (SD) increase in the PRS of antidepressant or lithium response, adjusting for the first four principal components (PCs). We ran these models in all phenotype comparisons. The proportion of variance in MDD treatment resistance explained by PRS (Nagelkerke’s R^2^) was calculated by comparing the full model including both PRS and covariates to the baseline model only including covariates. We converted Nagelkerke’s R^2^ to the liability scale by assuming 10% MDD cases meeting our stringent definition of TRD. We tested the trend of association in PRS quartiles with treatment resistance using Chi-squared test.

We employed a Bonferroni-corrected p-value threshold for statistical significance, controlling for multiple testing across six comparisons (two PRS of treatment response in three sets of TRD/non-TRD comparisons; P ≤ 0.05/6 = 0.008). All analyses were conducted in R (Version 4.0.0)^40^.

Sensitivity analysis. To account for potential genetic overlap between treatment response and corresponding psychiatric disorder risk (MDD and bipolar disorder)^12, 41^, we additionally adjusted for the PRS of MDD and bipolar disorder in the main models.

To examine whether the results were driven by lithium use in TRD patients, we further excluded patients with lithium use, and estimated the association between PRS of lithium response and treatment resistance in MDD.

## Results

After QC, 1 778 MDD cases who had received ECT and 2 264 without ECT were available for analyses. We further utilized the prescription records of antidepressants before the first ECT treatment to de ne TRD. Among the MDD cases with ECT (“*broad TRD*” definition), 1 674 cases (94.2%) had used antidepressants before the first ECT. The vast majority of these cases—1 487 (83.6%) and 1 081 (60.8%), respectively—had at least one or two different antidepressants of adequate duration (“*narrow_1 TRD*” and “*narrow_2 TRD*”), suggesting that these MDD cases receiving ECT were resistant to antidepressants. Among the 2 264 MDD cases without ECT (“*broad non-TRD*”), 1 483 (66.5%) had no more than two antidepressants of adequate duration (“*narrow non-TRD*”) (Table 1).

To investigate the genetic overlap between treatment response and resistance in MDD, we calculated the PRS of antidepressants and lithium response, and evaluated their associations with treatment resistance by comparing the PRS burdens among TRD and non-TRD cases.

With the polygenic component underlying antidepressants response^10^, we would expect an inverse association between the PRS of antidepressants response and treatment resistance in MDD. For the *broad* and *narrow_1* definitions, the results were in the expected effect direction, *i.e*., TRD cases had lower PRS of antidepressants response compared with non-TRD cases, although the mean differences were not significant (Table 1). Under these two definitions, per SD increase in the PRS of antidepressants response had a slightly reduced risk of TRD (e.g., OR = 0.98, 95% CI = 0.92–1.06, P = 0.67 under the *narrow_1* definition; *Table S1*). However, the effect direction was inconsistent in the *narrow_2* definition, potentially due to the reduced sample size in this most stringent definition. The results remained unchanged after further adjusting for the PRS of MDD (*Table S1*).

**Figure 1:**
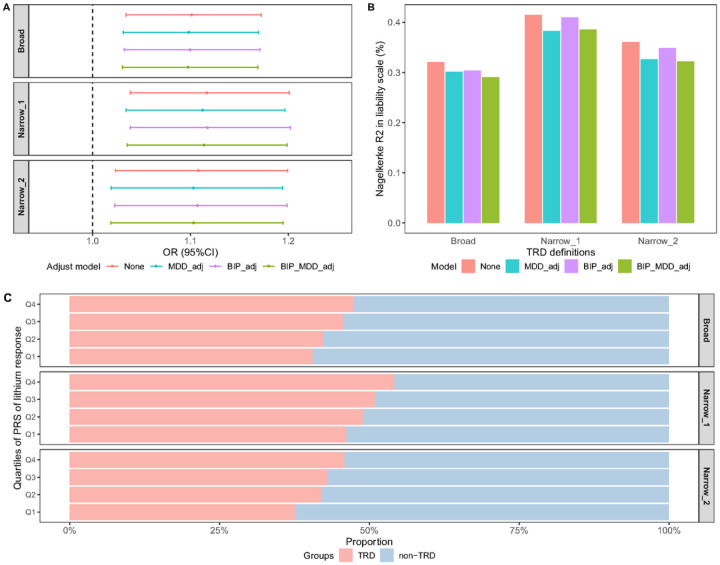
The results of lithium response PRS and TRD. (A) The association between PRS of lithium response and TRD. ORs for TRD associated with per-SD increase in the PRS of lithium response. Each panel shows ORs under different TRD definitions (see Materials and methods). (B) The proportion of variance in MDD treatment resistance explained by PRS of lithium response (Nagelkerke’s R^2^ on the liability scale). (C) The proportion of MDD cases (TRD and non-TRD) in each quartile (Q1-Q4) of the PRS of lithium response. Each panel represents one definition of TRD and non-TRD (see Materials and methods). The P value for trend analysis was 0.0006, 0.0015 and 0.0036 under broad, *narrow_1* and *narrow_2* definition, respectively. “None”: model adjusted only for the first four PCs. | “MDD_adj”: model additionally adjusted for the PRS of MDD. | “BIP_adj”: model additionally adjusted for the PRS of bipolar disorder. | “BIP_MDD_adj”: model additionally adjusted for the PRS of both MDD and BIP.

Given that lithium is recommended as an augmentation therapy for MDD patients who have experienced insufficient response to first-line antidepressants, we further tested the association of the lithium response PRS with MDD treatment resistance. TRD cases had significantly higher PRS of lithium response than non-TRD cases (mean difference = 0.11, P = 0.004 under *narrow_1* definition; Table 1). We found significant associations between PRS of lithium response and all TRD definitions in the logistic regression model, with per SD increase in the lithium response PRS associated with OR = 1.12 (95%CI = 1.04–1.20, P = 0.003 under the *narrow_1* definition; Fig. 1A, Table S1), although the variance explained was small (Fig. 1B). Further adjustment for additional PRS of MDD, bipolar disorder, or both, did not change the estimates. Similar results were observed after excluding TRD cases with lithium use (N = 502, 454 and 357 under broad, *narrow_1* and *narrow_2* definitions), suggesting that the association was not due to effect of lithium use among TRD cases (*Table S2*). To further quantify the effect of polygenic load of lithium response among MDD, we divided the MDD cases into quartiles based on their PRS of lithium response, and observed a clear trend of higher proportion of TRD cases in the higher PRS quartiles (P_*trend*_ <0.005 in all definitions; Fig. 1C). Taken together, the results suggested that TRD cases have higher polygenic load of responding to lithium compared to the non-TRD cases.

## Discussion

Leveraging the latest GWAS of treatment response and unique resources of clinically ascertained cohorts with comprehensive treatment records, we investigated whether polygenic load of treatment response differ between TRD and non-TRD cases. We found that compared to non-TRD, patients with TRD tend to have lower genetic load of antidepressant response and significantly higher genetic load of lithium response. These results provide evidence for the genetic overlap of treatment response with treatment resistance in MDD and reveal *an overall genetic pro le of antidepressant non-responsiveness and lithium-sensitivity in patients with TRD*.

To the best of our knowledge, this is the first study using the detailed treatment records of both antidepressants and ECT to derive definitions of TRD and non-TRD. Recent reviews highlighted the key outstanding issue in the research of psychiatric treatment resistance regarding its definition^14, 19, 20^. Indeed, three core components—correct diagnosis, adequate treatment, and non-response—are required to establish treatment resistance^14^. Data from clinical trials are useful for this purpose; however, there is a paucity of clinical trials with sufficient size and adequate length of follow-up. Recently, emerging efforts utilizing Electronic Health Records provide an exciting opportunity to study treatment response and resistance in large-scale, real-world healthcare settings^16, 42^. Using comprehensive clinical records from the Swedish healthcare registers, our work extends these efforts and further demonstrates that integrating the information of ECT use can provide a useful TRD definition. ECT is used to treat individuals with TRD^21, 22^, and we also used antidepressant prescription records to confirm that these individuals had multiple trials of adequate antidepressant drugs prior to ECT. Notably, our TRD definitions based on ECT and antidepressant use are more stringent and likely to ascertain more severe patients with TRD compared to those fulfill common TRD definitions based on failed antidepressants trials. One key advantage of using a stringent definition is to minimize misclassification between TRD and non-TRD groups which is common in the categorical-based definitions with certain cutoffs^43^. In addition, ascertaining severe TRD samples can be useful in particular for research purposes, *e.g*., extreme phenotyping has been shown as a powerful strategy for novel genetic discoveries^24, 44^. Finally, our definitions fit better with the recently proposed concept of “difficult to treat depression” (DTD). The DTD concept differs from the conventional TRD concept in that it moves away from the exclusive focus on acute symptomatic response and instead aims to reduce depression burden despite usual treatment efforts^20, 45, 46^.

Our work extended previous genetic studies of treatment response and further demonstrated the genetic overlap between treatment response and resistance in MDD. As expected, TRD cases appeared to have lower genetic load of responding to antidepressants compared with non-TRD, although the mean difference was non-significant. Comparing results under different definitions, it appeared that the “*broad*” and “*narrow_1*” definitions were capable of distinguishing between TRD and non-TRD cases. Similar results were found in the Generation Scotland study (177 TRD vs. 2,455 non-TRD; expected effect direction but also non-significant) using TRD definition with at least two switches between antidepressants^10^, confirming that our stringent definitions yielded consistent findings as other definitions commonly used in the literature. Further comparisons across different TRD definitions are warranted.

Lithium is known to be effective as an augmentation for patients with TRD^5, 47^. Our novel finding based on PRS, where TRD cases had notably higher genetic load to respond to lithium than non-TRD cases, adds to the evidence of lithium efficacy in treating TRD and further demonstrates that its effect is underpinned by genetic mechanisms. The mechanisms of action of lithium are unclear; however, earlier hypothesis suggested that it plays a role in 5-HT neurotransmission, and SNPs in the 5-HT transporter gene have been correlated with response to lithium in patients with depression^12, 48–51^. If the results are replicated, this finding will motivate future genetic studies to reveal mechanisms of actions involved in lithium.

This study features a stringent measure of MDD treatment resistance, along with the use of the largest GWAS to-date of treatment responses and unique clinical cohorts to investigate the genetics of treatment response and resistance in MDD. We examined response to antidepressants and lithium, but could not evaluate antipsychotics response here. Similar to lithium, atypical antipsychotics are also recommended as augmentation in MDD patients non-responding to antidepressants^52^. Previous GWAS have been conducted on antipsychotic response or resistance, although few specifically targeted the atypical antipsychotics used in MDD and with sufficient sample size^53, 54^. However, given that the effect of antipsychotic augmentation in TRD is similar to that of lithium^55^, we might expect similar findings for antipsychotic response. In addition, we lacked data on psychotherapies and other non-pharmacotherapies to integrate them in our TRD definition. This is a common issue when defining treatment resistance in psychiatric disorders^20^. But given that psychotherapy is used as first-line (before or combined with antidepressant use), it is less likely to impact our stringent TRD definition based on ECT use. Further efforts to incorporate multiple therapies are needed to re ne treatment resistance phenotype^9^.

In summary, we derived a stringent TRD definition based on antidepressant and ECT use to capture severe MDD with treatment resistance. Our results highlighted that patients with TRD have significantly higher genetic load of lithium response compared to non-TRD. This finding forms a genetic explanation for the effectiveness of lithium in treating TRD patients.

## Figures and Tables

**Table 1:** Sample size and mean differences in PRS of antidepressant and lithium response.

Definition	Sample sizes	Mean difference PRS_*antidepressant-response*_	Mean difference PRS_*lithium-response*_
Broad	1778 TRD, 2264 non-TRD	−0.015 (P = 0.631)	0.094 (P = 0.003)*
Narrow_1	1487 TRD, 1483 non-TRD	−0.010 (P = 0.794)	0.107 (P = 0.004)*
Narrow_2	1081 TRD, 1483 non-TRD	0.013 (P = 0.742)	0.104 (P = 0.009)

* Significance P < 0.008 after correcting for multiple comparisons.

## Data Availability

R scripts used for phenotype derivation and statistical analysis can be shared upon request.
